# Factors associated with infibulation among girls who underwent female genital mutilation in Guinea: Analysis DHS 2018

**DOI:** 10.4102/jphia.v16i1.1280

**Published:** 2025-08-08

**Authors:** Kaba S. Keita, Tiany Sidibe, Alpha O. Sall, Sadan Camara, Fanta Barry, Ramata Diallo, Madeleine Toure, Aissatou Diallo, Mamadou D. Balde, Alexandre Delamou

**Affiliations:** 1 Department of Public Health, Centre for Research in Reproductive Health in Guinea (CERREGUI), Conakry, Guinea; 2 African Centre of Excellence for Prevention and Control of Transmissible Diseases (CEA-PCMT), University Gamal Abdel Nasser, Conakry, Guinea; 3 Department of Public Health, Maferinyah National Centre for Training and Research in Rural Health (CNFRSR), Conakry, Guinea

**Keywords:** infibulation, associated factors, FGM/C, DHS, Guinea

## Abstract

**Background:**

Female genital mutilation (FGM), especially infibulation, is a significant public health issue that poses numerous health risks for young girls. Despite its severity, this phenomenon remains under-documented.

**Aim:**

This study aims to identify the factors associated with infibulation among girls who underwent FGM in Guinea.

**Setting:**

This study was conducted in Guinea.

**Method:**

A secondary analysis of data from the 2018 Demographic and Health Survey (DHS) in Guinea included 3950 women whose daughters had undergone female genital mutilation or excision (FGM/E). A multivariate logistic regression was performed to identify factors associated with infibulation using the Stata software version 17.

**Results:**

The overall prevalence of infibulation among girls who underwent female genital mutilation or cutting (FGM/C) in Guinea was 16%. This prevalence was higher at 17% (95% confidence interval [CI]: [0.1–0.2]) among girls aged 0 years to 4 years. Statistically significant individual and contextual factors included: maternal age (odds ratio [OR] = 1.4, 95% CI: [1.1–2.6]), maternal employment status (OR = 1.7, 95% CI: [1.3–2.2]), maternal religion (OR = 2.7, 95% CI: [1.2–5.8]), maternal infibulation status (OR = 22.1, 95% CI: [16.6–29.4]) and region of residence (OR = 2.8, 95% CI: [1.6–4.8]).

**Conclusion:**

This study highlights the need for educational, socio-economic and public health strategies to eradicate infibulation in Guinea and promote sustainable change.

**Contribution:**

This research revealed the influence of individual and contextual factors on infibulation and highlighted the emergency of targeted strategies, such as awareness raising, community dialogue and education about its risks.

## Background

Female genital mutilation or cutting (FGM/C), also known as female circumcision, is a public health and human rights issue affecting women and girls.^1^ In 1997, the World Health Organization (WHO), the United Nations Children’s Fund (UNICEF) and the United Nations Population Fund issued a joint statement defining FGM/C as any procedure involving partial or total removal of the external female genitalia or other injury to the external female genitalia.^2^ The female genitalia, whether for cultural, religious or other non-therapeutic reasons, sometimes undergo changes.^3,4^ An estimated 200 million girls and women in 30 countries, primarily in Africa, the Middle East and Asia have experienced the practice.^5,6^ Around 3 million girls are at risk of being excised each year.^4^ Female genital mutilation or cutting has been associated with a wide range of medical, economic and sociocultural consequences.^7^ In Africa, the prevalence of FGM/C is still high despite declining over the past three decades. Prevalence has decreased from 57.7% in 1990 to 14.1% in 2015 in North Africa, from 71.4% in 1995 to 8.0% in 2016 in East Africa and from 73.6% in 1996 to 25.4% in 2017 in West Africa.^8,9,10^ Female genital mutilation or cutting in Africa is a practice that has persisted because of strong sociocultural influences that ensure that it is carried out in secret and under-reported. For decades, it has been a practice that has been adopted by all major religions prevalent on the continent, including Christianity, Islam and traditional religion.^11^

In most communities, FGM is seen as a means of ensuring women’s sexual morality.^12^ Mothers of girls aged from 0 years to 14 years old reported that 35% of them had been excised by a health care provider. The rate of medicalisation is twice as high in this age group as in adult women, implying a growing trend towards medicalised FGM.^13,14^ Infibulation serves as a significant symbol closely associated with the physical nature of the procedure. The opening left in the infibulated scar must be small enough to prevent sexual intercourse, thereby fulfilling its primary roles of protection and proof of virginity. Traditionally, only an infibulated girl was recognised as a virgin, considered a virtuous and respectable woman in society.^15,16^ Most of the reported complications of FGM are related to the most severe form which is infibulation.^17^ A woman without infibulation, on the other hand, would traditionally be considered lacking in boundaries, self-control or respect. She would be seen as accessible to sexual advances from any man. She would be considered an immoral woman who could never be trusted with the paternity of her children.^18,19^ This situation could threaten the social structure built on paternal clan affiliation.^20^ For these reasons, a woman without infibulation would not be considered worthy of marriage.^15,20^ There is variation in the prevalence of infibulation within the African countries that practise it. In Sudan, Somalia^21,22^ and Djibouti,^23^ the prevalence of infibulation among girls is greater than 80%. In Eritrea, Ethiopia, Kenya, Mali, Senegal and Niger,^23^ the prevalence ranges from 10.2% to 38.6%. Low prevalences ranging from 1.2% to 8.7% have been recorded in Burkina Faso, Benin, Côte d’Ivoire, Sierra Leone, Nigeria and Chad.^23^ In Guinea, the practice of infibulation is a little more widespread in rural than in urban areas (17% against 13%). When mothers are infibulated, their daughters are more likely to undergo the procedure. In fact, among infibulated mothers, 56% of excised daughters had their vagina sewn shut, compared with 11% of excised daughters whose mothers were excised but not infibulated.^14^ However, the true prevalence of infibulation is likely to be even higher because there is a general tendency to underreport.^24^ Several studies have focused on FGM in general and have identified factors associated with this practice. However, most of these studies used cross-sectional approaches and systematic reviews of FGM in general.^8,9,25,26,27,28,29,30,31^ As a result, there is little data on the practice of infibulation, including the factors associated with this phenomenon. However, these individual and contextual factors are important for understanding the phenomenon, which has numerous consequences for the health of young girls. The objective of this study was to identify individual and contextual factors of the practice of infibulation among girls who underwent FGM/C in Guinea.

## Methods

### Conceptual framework

**FIGURE 1:**
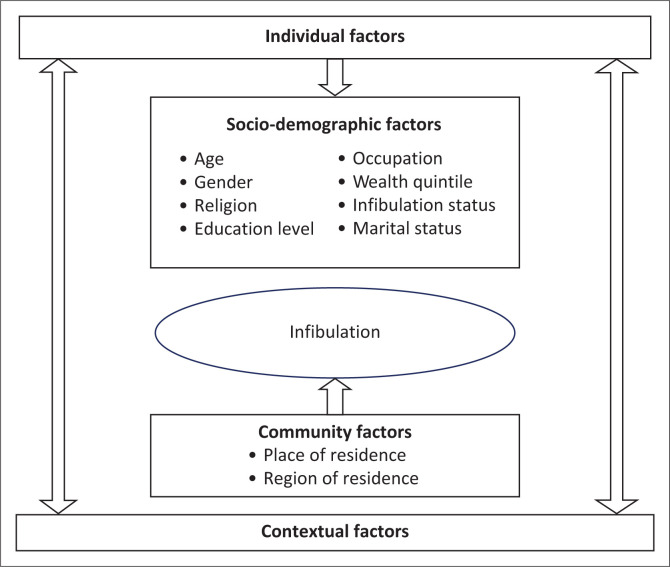
Conceptual framework of factors involved in the infibulation process.

Our conceptual framework was inspired by the theory of social conventions and norms,^32^ which seems to have influenced the practice of infibulation. The practice of infibulation is rooted in gender roles, community influence and a sense of belonging. It is not a religious practice, but a cultural one that reflects shared values concerning family honour, feminine virtues, sexual abstinence and other codes of conduct. The practice of infibulation continues because of custom, belief, social pressure, patriarchy and the assumption that it increases girls’ chances of marriage. However, it is practised to control girls’ bodies and sexuality for the benefit of men, as it is perceived as guaranteeing virginity until marriage. It is passed down from older to younger generations as an obligation accepted by the community. The practice is a symbol of social control over female sexual pleasure and is linked to women’s reproductive roles in society. It is also thought to increase sexual pleasure in men and make women attractive. Social norm theory has become important for understanding why infibulation continues and for designing interventions to end the practice. It is possible to design interventions to accelerate the abandonment of infibulation without undermining traditional values. The present study will investigate the individual and contextual factors (Figure 1) associated with the practice of infibulation among girls aged 0–14 years in Guinea.

### Study design

This was a secondary analysis of data from the 2018 Demographic and Health Survey (DHS) of Guinea. The primary study was a cross-sectional survey of women of reproductive age with daughters aged from 0 years to 14 years old who had undergone FGM. Data collection took place from March 2018 to June 2018 through face-to-face interviews by trained data collectors to standardise understanding and administration of the questionnaire.

### Study setting

The Republic of Guinea is located in West Africa, between 7° and 12° north latitude and 8° and 15° west longitude. It is bordered by Guinea Bissau to the northwest, Senegal and Mali to the north, Côte d’Ivoire to the east, Liberia and Sierra Leone to the south and the Atlantic Ocean to the west. The population was estimated at 13 million inhabitants in 2018, of which 51.5% were women. Administratively, Guinea is made up of 7 administrative regions, plus the city of Conakry, which has the status of a decentralised community. The country has 33 prefectures, 38 urban communes, including five in Conakry and 334 rural communes. These communities are subdivided into 308 urban neighbourhoods and 1615 rural districts. The public health system is organised into three levels: the first level, comprising 407 rural and urban health centres, 26 district hospitals and 9 communal medical centres; the second level, comprising eight regional hospitals; and the third level, represented by three national hospitals.^33^

### Study population

The study population consisted of all girls aged from 0 years to 14 years old who underwent female genital mutilation or excision (FGM/E), and residing on the national territory at the time of the survey. In total, the primary survey covered 3950 women with at least one daughter who has undergone FGM.

### Source and extraction of data

For this study, secondary data from the 2018 Guinea DHS were used. These data are accessible in the DHS programme repository (https://dhsprogram.com/data/available-datasets.cfm)^33^ to all registered users and can be downloaded with permission. After reviewing and understanding the details of the data structure, the appropriate data type for the analysis of female infibulation was selected. For this study, the file that contains data for women of reproductive age (15–49 years old) was used. A sample of 3950 women who had excised daughters with infibulation was considered. Data on infibulation and its potential explanatory variables (individual and contextual level variables) were extracted.

### Study variables

#### Dependent variable

Female infibulation was considered in this study as the outcome variable. It was measured by asking women who have at least one excised daughter whether the excised daughter was infibulated. A binary outcome was created from this response. If the daughter was infibulated, the infibulation status was coded as ‘Yes (1)’ and if the daughter was not infibulated, the infibulation status was coded as ‘No (0)’.

#### Independent variables

Individual and contextual or community variables were included as potential explanatory variables for daughter infibulation. Individual-level variables include individual socio-demographic characteristics, while contextual-level variables include common characteristics of the study subjects based on enumeration areas in the DHS-2018 data. In this study, 15 explanatory variables, composed of 13 individual factors and 2 contextual factors were listed.

Household wealth quintiles (in the standard DHS, wealth quintile is calculated from data on household ownership of selected assets such as television, bicycle and car; housing characteristics such as materials used in house construction; type of drinking water source; toilet; electricity; sanitation facilities and other characteristics). A composite variable, wealth status, is created from these assets through principal component analysis (PCA) by placing households on a continuous measure of relative wealth, after which households are classified into five wealth quintiles ‘poorest, poor, middle, rich and richest’. We recoded these wealth quintiles into three categories namely ‘Poor (1), Middle (2) and Rich (3)’.

### Data processing

Additional recording of variables was carried out as needed for the analysis. The data being processed and cleaned were analysed using Stata Version 17, and QGIS/GIS 3.10 software was used for spatialisation of infibulation data. Out of a sample of 3950 excised girls, 21 (0.53%) were excluded because of missing data at the time of modelling. Model specification was performed using the Hosmer-Lemeshow test while multicollinearity was tested using the variance inflation factor (VIF). Sampling weights were applied to correct and account for the sampling structure, while the SVY command (in Stata is used to analyse complex survey data: stratification, clusters, etc.) was used to address the complex nature of the survey and generalise the results.

### Data analysis

The analyses consisted of basic descriptive statistics to describe the data and characteristics of the respondents as well as the calculation of the prevalence of infibulation in girls aged from 0 years to 14 years old. The Chi-square test of independence was used to assess the statistical significance of the association between each of the explanatory variables and infibulation in girls aged from 0 years to 14 years old. Variables that showed significant associations with infibulation at the Chi-square level at a p-value less than 0.20 and variables that were considered to have an association with infibulation through the literature review were used in the logistic regression modelling. The results of the regression analysis were presented as crude odds ratios (bivariate analysis) and adjusted odds ratios with their p-value and 95% confidence interval (CI) indicating precision. For the validation of the final model, the estimation quality measures and influential values were used as post-estimation tests (likelihood ratio test, model discriminatory power, global model fit, model diagnostics, etc.).

### Ethical considerations

The primary study had obtained authorisation from the Health Research Ethics Committee and, in our analyses, we respected the confidentiality of the participants. As the data were not collected by the authors of this study, we requested authorisation from the Demographic and Health Surveys (DHS) Programme to exploit the DHS-2018 data, and access to the data was provided after our intention of the request was assessed and approved. The dataset is freely available at (https://dhsprogram.com/data/available-datasets.cfm).

## Results

### Description of the study population according to individual and contextual characteristics

The study focused on 3950 girls who have undergone FGM residing in all regions of Guinea. Table 1 presents the individual and contextual characteristics of the study population. The average age of the mothers was 35.5 (7.1) years old and that of the girls was 7.5 (13.9) years old. The male gender of the head of household was the most represented in the sample (87.6%). Mothers aged from 35 years to 44 years old represented 42% and more than half (58.5%) of the infibulated girls were aged from 5 years to 9 years old. The majority of mothers were married (95.5%), without any level of education (87.7%). More than a third (34%) of them had five to six living children and 93.8% practised the Muslim religion. More than 11% of mothers were infibulated, while 15% of them did not know their infibulation status. About 46% of mothers lived in a household with a poor wealth index and 74.5% resided in rural areas.

**TABLE 1:** Socio-demographic characteristics of women with daughters aged 0 years to 14 years who have undergone female genital mutilation in Guinea, 2018.

Variables	Total (*n*)	Percentage
**Factors at the individual level**		
Gender of household’s head	3950	-
Male	3461	87.6
Female	489	12.4
Girl’s age (in years)	3950	-
0–4	1277	32.3
5–9	2310	58.5
10–14	363	9.2
Mother’s age (in years)	3950	-
15–24	203	5.1
25–34	1559	39.5
35–44	1646	41.7
≥ 45	541	13.7
Age of household’s head (in years)	3950	-
15–34	374	9.5
35–44	1040	26.3
45–54	1158	29.3
≥ 55	1378	34.9
Mother’s marital status	3950	-
Married	3772	95.5
Single	40	1.0
Widow, divorced or separated	138	3.5
Number of mother’s living children	3 950	-
1–2	380	9.6
3–4	1206	30.5
5–6	1334	33.8
≥ 7	1030	26.1
Mother’s current occupation	3950	-
No	1095	27.7
Yes	2855	72.3
Husband’s current occupation	3764	-
No	276	7.3
Yes	3488	92.7
Mother’s religion	3950	-
Muslim	3705	93.8
Christian	245	6.2
Mother’s educational level	3950	-
Not in school	3464	87.7
Primary	221	5.6
Secondary	221	5.6
Higher	44	1.1
Husband’s educational level	3764	-
Not in school	2996	79.6
Primary	237	6.3
Secondary	354	9.4
Higher	177	4.7
Mother’s infibulation status	3929	-
No	2885	73.4
Yes	448	11.4
Don’t know	596	15.2
Wealth index	3950	-
Poor	1801	45.6
Middle class	865	21.9
Wealthy	1284	32.5
**Contextual level factor**		
Residential environment	3950	-
Urban	1007	25.5
Rural	2943	74.5
Region of residence	3950	-
Boké	502	12.7
Conakry	403	10.2
Faranah	336	8.5
Kankan	766	19.4
Kindia	668	16.9
Labé	521	13.2
Mamou	446	11.3
N’Zérékoré	308	7.8

*Source:* National Institute of Statistics. ICF. Guinea Demographic and Health Survey (DHS V) 2016-18. Conakry: INS/Guinea and ICF; 2019

### Prevalence of infibulation among girls who underwent female genital mutilation or excision

The overall prevalence of infibulation among girls who underwent FGM/C in Guinea was 16% (95% CI: [0.1–0.2]).

This prevalence was 17% (95% CI: [0.1–0.2]) among girls aged from 0 to 4 years old and 14% (95% CI: [0.1–0.1]) and 15% (95% CI: [0.1–0.2]) for the other two age groups.

**FIGURE 2:**
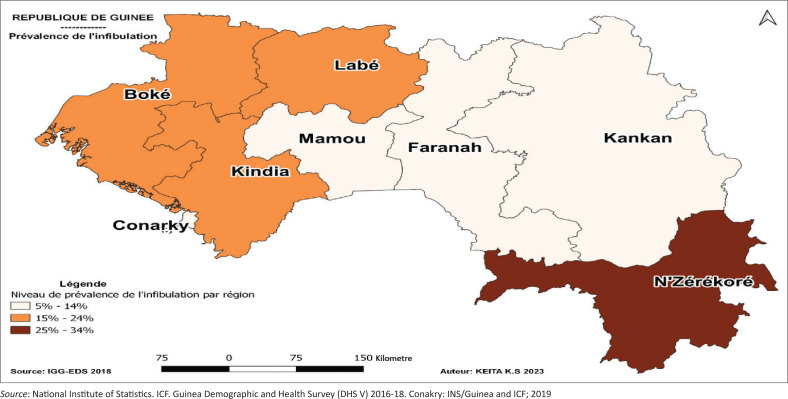
Mapping the prevalence of infibulation by region, 2018 Demographic and Health Survey, Guinea.

There is a variation in the distribution of this prevalence according to the place of residence. It was 13% (95% CI: [0.1–0.2]) in urban areas and 16% (95% CI: [0.1–0.2]) in rural areas. As for the distribution of prevalence according to regions of residence (Figure 2), it was 31% (95% CI: [0.2–0.4]) in N’Zerekore; 23% (95% CI: [0.2–0.3]) in Boké; 16% (95% CI: [0.1–0.2]) in Labé; 15% (95% CI: [0.1–0.2]) in Kindia; 14% (95% CI: [0.1–0.2]) in Faranah; 12% (95% CI: [0.1–0.2]) in Conakry; 11% (95% CI: [0.1–0.2]) in Kankan and 8% (95% CI: [0.1–0.2]) in Mamou.

### Factors associated with infibulation among girls who underwent female genital mutilation or excision in Guinea 2018 in bivariate and multivariate analysis

In the bivariate analysis, the variables significantly associated with infibulation among girls who underwent FGM/C included the age of the head of household, age of the mother, number of living children of the mother, current occupation of the mother, religion of the mother, education level of the husband, infibulation status of the mother, wealth index, area of residence and region of residence. In multivariate analysis, four individual factors and one contextual factor were significantly associated with infibulation among girls who underwent FGM/C namely: age of the mother, current occupation of the mother, religion of the mother, infibulation status of the mother and region of residence (Table 2).

**TABLE 2:** Factors associated with infibulation among girls aged 0 years to 14 years who underwent female genital mutilation/cutting in Guinea in multivariate analysis (*N* = 3950).

Variables	Factors associated with infibulation					
	Crude OR	95% CI	*p*	Adjusted OR	95% CI	*p*
**Age of household’s head (in years)**						
15–34	1.8***	**1.3–2.5**	**< 0.001**	1.5	0.9–2.3	0.066
35–44	1.0	0.8–1.3	0.738	1.2	0.9–1.7	0.214
45–54	1.0	0.8–1.3	0.964	1.1	0.8–1.5	0.423
55 +	Ref	-	-	Ref	-	-
**Mother’s age (in years)**						
15–24	1.4	0.9–2.4	0.157	1.7	0.8–3.7	0.185
25–34	1.4*	**1.1–1.9**	**0.015**	1.7*	**1.1-2.6**	**0.012**
35–44	1.3	0.9–1.7	0.100	1.4*	**1.0-2.1**	**0.047**
≥ 45	Ref	-	-	Ref	-	-
**Number of mother’s living children**						
1–2	1.1	0.9–1.6	0.491	0.9	0.5–1.5	0.613
3–4	1.4*	**1.1–1.8**	**0.016**	1.0	0.7–1.4	0.816
5–6	0.9	0.7–1.2	0.383	0.8	0.6–1.1	0.140
≥ 7	Ref	-	-	Ref	-	-
**Mother’s current occupation**						
No	1.5***	**1.3–1.9**	**< 0.001**	1.7***	**1.3–2.2**	**< 0.001**
Yes	Ref	-	-	Ref	-	-
**Husband’s current occupation**						
No	0.7	0.5–1.0	0.075	0.8	0.5–1.4	0.530
Yes	Ref	-	-	Ref	-	**-**
**Mother’s religion**						
Muslim	Ref	-	-	Ref	-	**-**
Christian	3.6***	**2.5–5.3**	**< 0.001**	2.7*	**1.2–5.8**	**0.015**
**Mother’s educational level**						
Not in school	1.3	0.4–3.7	0.667	1.7	0.2–12.7	0.581
Primary	1.0	0.3–3.3	0.935	1.7	0.2–12.8	0.611
Secondary	0.8	0.2–2.5	0.669	1.6	0.2–12.3	0.655
Higher	Ref	-	-	Ref	-	-
**Husband’s educational level**						
Not in school	1.9*	**1.1-3.2**	**0.017**	1.3	0.7–2.7	0.399
Primary	1.5	0.8–3.0	0.188	0.9	0.4–2.0	0.726
Secondary	2.4**	**1.3–4.5**	**0.004**	1.6	0.7–3.3	0.223
Higher	Ref	-	-	Ref	-	-
**Mother’s infibulation status**						
No	Ref	-	-	Ref	-	-
Yes	19.7***	**15.2–25.6**	**< 0.001**	22.1***	**16.6–29.4**	**< 0.001**
Don’t know	7.4***	**5.6–9.6**	**< 0.001**	7.7***	**5.8–10.2**	**< 0.001**
**Wealth Index**						
Poor	1.6***	**1.3–2.1**	**< 0.001**	1.2	0.8–1.8	0.432
Middle class	1.5**	**1.1–2.0**	**0.004**	1.4	0.9–2.2	0.147
Wealthy	Ref	-	-	Ref	-	-
**Place of residence**						
Urban	Ref	-	-	Ref	-	-
Rural	1.3*	**1.1–1.7**	**0.012**	0.9	0.5–1.3	0.532
**Region of residence**						
Boké	2.2***	**1.4–3.2**	**< 0.001**	2.8***	**1.6–4.8**	**< 0.001**
Conakry	Ref	-	-	Ref	-	-
Faranah	1.2	0.7–1.9	0.456	1.2	0.7–2.2	0.512
Kankan	0.9	0.5–1.4	0.549	1.8	0.9–3.2	0.060
Kindia	1.3	0.9–2.0	0.195	1.4	0.8–2.4	0.206
Labé	1.3	0.9–2.0	0.187	1.1	0.6–2.0	0.750
Mamou	0.7	0.4–1.1	0.110	0.7	0.4–1.3	0.239
N’Zérékoré	3.3***	**2.0–5.4**	**< 0.001**	1.3	0.5–3.2	0.512

*Source:* National Institute of Statistics. ICF. Guinea Demographic and Health Survey (DHS V) 2016-18. Conakry: INS/Guinea and ICF; 2019

OR, odds ratio; CI, confidence interval; Ref, reference.

* , Significance at *p* < 0.05;

** , Significance at *p* < 0.01;

*** , Significance at *p* < 0.001.

Table 2 presents the factors associated with infibulation among girls who underwent FGM/C in multivariate analysis. Girls whose mothers were aged from 25 years to 34 years and 35–44 years old had, respectively, 1.7 (OR = 1.7, 95% CI: [1.1–2.6]) and 1.4 (OR = 1.4, 95% CI: [1.0–2.1]) times higher odds of being infibulated compared to girls whose mothers were 45 years and older. Girls whose mothers were in an income-generating activity were 1.7 (OR = 1.7, 95% CI: [1.3–2.2]) times more likely to undergo infibulation compared to those whose mothers were not in an income-generating activity. By religion, Christian mothers were 2.7 (OR = 2.7, 95% CI: [1.2–5.8]) times more likely to have infibulated daughters compared to Muslim mothers. Daughters whose mothers were infibulated were 22.1 times more likely to undergo infibulation compared to those whose mothers were not infibulated (OR = 22.1, 95% CI: [16.6–29.4]). In contrast, daughters whose mothers did not know their infibulation status compared to those whose mothers were not infibulated were 7.7 (OR = 7.7, 95% CI: [5.8–10.2]) times more likely to be infibulated. Compared to girls residing in the region of Conakry, those living in the region of Boké were 2.8 (OR = 2.8, 95% CI: [1.6–4.8]) times more likely to undergo infibulation.

## Discussion

The aim of this study was to identify the main explanatory factors for the practice of infibulation among girls who underwent FGM/C in Guinea. The results of this study show a high prevalence of infibulation in Guinea and identify four individual and contextual factors significantly associated with its practice. These results have important implications for the fight against the practice of infibulation in Guinea.

Sixteen out of 100 girls were victims of infibulation in Guinea in 2018. This prevalence is significantly higher than those reported in Burkina Faso (1.2%) in 2010^34^ and in Chad (3.5%) in 2014–2015.^35^ This difference with our results could be explained by the specificity of the study population. However, it is lower than the estimates made by Farage et al.^23^ in Somalia (36.1%), Sudan (82.4%) and Eritrea (38.6%). A possible explanation could be the difference in the cultural background of the study participants.

To our knowledge, there is little information on the factors associated with infibulation among girls, with available studies being limited to factors associated with FGM in general. In this study, the age of adult mothers was found to be a factor in increasing the practice of infibulation among girls. This could be justified by a strong medicalisation of the practice of infibulation. Women of childbearing age have more access to health services to perform infibulation in hygienic conditions than older women. Similarly, with the older generation, it is likely that dangers related to ingrained cultural norms decrease the practice of infibulation. We found in the study that compared to girls whose mothers were engaged in an income-generating activity, a higher probability of infibulation was observed among girls whose mothers did not have an income-generating activity. Contrary to our results, a study conducted by Ahinkorah BO in Chad in 2021^25^ had shown that compared to working women, a lower probability of FGM among girls was observed among non-working women (OR = 0.77, CI = 0.63–0.95). This result from our study can be explained by the fact that working mothers are often better educated and more aware of the risks associated with infibulation. They may also have better opportunities for communication and access to information, leading to a lower likelihood of performing infibulation on their daughters. Most often, mothers who are in paid employment are economically independent enough to make important household decisions, including entrenched cultural traditions and norms such as infibulation.

The results showed that compared to daughters of Muslim mothers, daughters of Christian mothers were 2.7 times more likely to undergo infibulation. Indeed, we could explain this finding by the fact that the practice of infibulation during early childhood in communities is considered part of cultural rites. Generally, in certain areas with a high prevalence of infibulation such as the region of Boké where the practice of the Christian religion is higher, a relative increase in this practice is observed.

In addition, this may be because of the fact that people of the same religion can share common characteristics in terms of certain traditional practices. The study found that girls whose mothers were infibulated had a higher probability of undergoing infibulation. Nnanatu et al.^36^ in a study on female genital mutilation in girls aged from 0 years to 14 years old in Nigeria reported that the probability of FGM was higher in girls, whose mothers underwent FGM. Indeed, the result observed in our study may be explained by the tendency of mothers who underwent infibulation at a young age to transmit this practice to their daughters during early childhood. It is also about preserving virginity, decreasing sexual desire and controlling the sexuality of girls until marriage.

Moreover, this may be because of the stronger tendency of infibulated mothers to support the continuation of infibulation than non-infibulated mothers. This finding shows that communities that practise infibulation on their daughters may also be at considerable risk of continuing the practice of infibulation persistently in the future. The region of residence of mothers also significantly affects the infibulation status of girls. Mothers residing in the region of Boké compared to their counterparts in the region of Conakry were 2.8 times more likely to have infibulated daughters. These findings may be related to the difference in local cultural background and suggest that infibulation may also be common among Christian practitioners. Cultural influences such as adherence to accepted community norms may explain this. This finding contrasts with an Ethiopian study on factors associated with FGM among daughters of women of reproductive age.^27^

### Strengths and limitations

Some limitations deserve to be highlighted in the context of the realisation of this study. Firstly, the DHS is a cross-sectional study. Although it allows for establishing associations between the practice of infibulation and various explanatory variables, it does not establish a causal link. Secondly, some information may introduce measurement bias, such as the infibulation status of the girl, as the data collection relied solely on the mother’s declaration. Although self-reported data are commonly used and can provide useful information, it is essential to consider the potential biases they introduce. To increase the reliability of results, researchers should consider adopting rigorous validation strategies, combining objective data and employing carefully designed, diversified collection methods. Thirdly, some independent variables such as the mother’s current job could lead to a selection bias. Finally, the lack of qualitative data limits the in-depth understanding and explanation of the models generally characterised by a quantitative analysis.

Despite these limitations, the strengths of the study lie in the use of nationally representative data and a large sample size that guarantees the reliability and generalisability of the results to all girls in Guinea. The study could contribute to improving understanding of factors related to the practice of infibulation among infibulated girls aged from 0 years to 14 years old in Guinea.

### Implications for research and practice

In Guinea, infibulated women are the most supportive of the infibulation of girls, and there is also great potential to educate and persuade this group about the importance of eliminating the practice. Given the continued and consistently high prevalence of infibulation over the decades and the controversial cultural mores associated with infibulation practices, it would be useful for the Ministry of Health to ensure the implementation of regulatory texts prohibiting the practice of infibulation in all health institutions. In addition, it would be crucial to adopt policies that strengthen the engagement of communities and religious leaders in the prevention and control of infibulation. Present a precise framework for campaign implementation, with clear stages, necessary resources, monitoring indicators and impact assessment mechanisms. This will help policy-makers understand how to translate recommendations into concrete action. Furthermore, in synergy with other policies, raising awareness and promoting behavioural change among populations could ultimately lead to ending the practice of infibulation in Guinea.

## Conclusion

This research identified factors associated with infibulation among girls aged from 0 years to 14 years old in Guinea, highlighting the significant influence of various individual and contextual factors, responsible for the practice of infibulation. Key factors identified include the age of the mother, the current occupation of the mother, the religion of the mother, the infibulation status of the mother and the region of residence. These findings highlight the emergency of developing targeted strategies and educational interventions, including media awareness, community dialogue, and promoting understanding of the risks and consequences of infibulation. It is imperative that relevant entities, such as the Ministry of Health of Guinea and reproductive health stakeholders, prioritise these factors in policy and programme development to consider significant improvement in the future.

## References

[1] Kandala NB, Nnanatu CC, Atilola G, et al. A spatial analysis of the prevalence of female genital mutilation/cutting among 0-14-Year-Old girls in Kenya. Int J Environ Res Public Health. 2019;16(21):4155. 10.3390/ijerph16214155 31661902 PMC6862646

[2] Goldberg H, Stupp P, Okoroh E, Besera G, Goodman D, Danel I. Female genital mutilation/cutting in the United States: Updated estimates of women and girls at risk, 2012. Public Health Rep. 2016;131(2):340–347. 10.1177/003335491613100218 26957669 PMC4765983

[3] WHO/UNICEF/UNFPA. Female genital mutilation: Statement [homepage on the Internet]. [cited 2025 Jan 15]. Geneva: WHO; 1997. Available from https://iris.who.int/bitstream/handle/10665/41903/9241561866.pdf?seq

[4] WHO. Eliminating female genital mutilation [homepage on the Internet]. [cited 2025 Jan 5] Geneva: OHCHR, UNAIDS, UNDP, UNECA, UNESCO, UNFPA, UNHCR, UNICEF, UNIFEM, WHO. 2008. Available from: https://iris.who.int/handle/10665/43839

[5] Alradie-Mohamed A, Kabir R, Arafat SMY. Decision-making process in female genital mutilation: A systematic review. Int J Environ Res Public Health. 2020;17(10):3362. 10.3390/ijerph17103362 32408674 PMC7277396

[6] UNICEF. Female genital mutilation/cutting : A global concern. New York: UNICEF; 2016.

[7] Muteshi JK, Miller S, Belizán JM. The ongoing violence against women: Female Genital Mutilation/Cutting. Reprod Health. 2016;13:44. 10.1186/s12978-016-0159-3 27091122 PMC4835878

[8] Ahinkorah BO, Hagan JE, Ameyaw EK, et al. Socio-economic and demographic determinants of female genital mutilation in sub-Saharan Africa: Analysis of data from demographic and health surveys. Reprod Health. 2020;17(1):162. 10.1186/s12978-020-01015-5 33092624 PMC7584098

[9] Geremew TT, Azage M, Mengesha EW. Hotspots of female genital mutilation/cutting and associated factors among girls in Ethiopia: A spatial and multilevel analysis. BMC Public Health. 2021;21(1):186. 10.1186/s12889-021-10235-8 33478450 PMC7818563

[10] Kandala NB, Ezejimofor MC, Uthman OA, Komba P. Secular trends in the prevalence of female genital mutilation/cutting among girls: A systematic analysis. BMJ Glob Health. 2018;3(5):e000549. 10.1136/bmjgh-2017-000549 PMC6231106 30483404

[11] Odukogbe ATA, Afolabi BB, Bello OO, Adeyanju AS. Female genital mutilation cutting in Africa. Transl Androl Urol. 2017;6(2):138–148. 10.21037/tau.2016.12.01 28540220 PMC5422681

[12] Berg RC, Denison E. A tradition in transition: Factors perpetuating and hindering the continuance of Female Genital Mutilation/Cutting (FGM/C) summarized in a systematic review. Health Care Women Int. 2013;34(10):837–859. 10.1080/07399332.2012.721417 23489149 PMC3783896

[13] Balde MD, O’Neill S, Sall AO, et al. Attitudes of health care providers regarding female genital mutilation and its medicalization in Guinea. PLoS One. 2021;16(5):e0249998. 10.1371/journal.pone.0249998 33983949 PMC8118326

[14] National Institute of Statistics Ministry of Planning and Economic Development Conakry. Guinea demographic and health survey 2018. Conakry (Guinea): ICF International; 2019.

[15] Gruenbaum E. Sexuality issues in the movement to abolish female genital cutting in Sudan. Med Anthropol Q. 2006;20(1):121–138. 10.1525/maq.2006.20.1.121 16612996

[16] Abdalla RHD. Sisters in affliction: Circumcision and infibulation of women in Africa. London: Zed Press; 1982, p. 122.

[17] Rushwan H. Female genital mutilation (FGM) management during pregnancy, childbirth and the postpartum period. Int J Gynecol Obstet. 2000;(70):99–104. 10.1016/S0020-7292(00)00237-X 10884538

[18] Berg RC, Underland V. The obstetric consequences of female genital mutilation cutting: A systematic review and meta-analysis. Obstet Gynecol Int. 2013;2013:496564. 10.1155/2013/496564 23878544 PMC3710629

[19] Banks E, Meirik O, Farley T, Akande O, Bathija H, Ali M. Female genital mutilation and obstetric outcome: WHO collaborative prospective study in six African countries. Lancet. 2006;367(9525):1835–1841. 10.1016/S0140-6736(06)68805-3 16753486

[20] Talle A. Transforming women into ‘Pure’ agnates: Aspects of female infibulation in Somalia. In *Carved flesh/cast selves*. 1st ed. Routledge; 2021, p. 83–106.

[21] Department of Statistics and Institute for Resource Development/Macro International Inc. Sudan Demographic and Health Survey 1989/1990. Khartoum: Institute for Resource Development/Macro International, Inc; 1991.

[22] Somaliland Ministry of Planning and National Development, UNICEF. Somaliland Multiple Indicator Cluster Survey 2011: Final Report, March 2014 : Monitoring the Situation of Children and Women. Hargeisa (Somaliland): Ministry of Planning and National Development and UNICEF; 2014.

[23] Farage MA, Miller KW, Tzeghai GE, Azuka CE, Sobel JD, Ledger WJ. Female genital cutting: Confronting cultural challenges and health complications across the lifespan. Womens Health (Lond Engl). 2015;11(1):79–94. 10.2217/WHE.14.63 25581057

[24] Crawford S, Ali S. Situational analysis of FGM/C stakeholders and interventions in Somalia. Oxford (UK): Health and Education Advice and Resource Team (HEART). 2015; p. 14–16.

[25] Ahinkorah BO. Factors associated with female genital mutilation among women of reproductive age and girls aged 0–14 in Chad: A mixed-effects multilevel analysis of the 2014–2015 Chad demographic and health survey data. BMC Public Health. 2021;21(1):286. 10.1186/s12889-021-10293-y 33541311 PMC7863379

[26] Allam MF, Irala EJ, Fernández CNR, et al. Factors associated with the condoning of female genital mutilation among university students. Public Health. 2001;115(5):350–355. 10.1038/sj.ph.1900791 11593445

[27] Gajaa M, Wakgari N, Kebede Y, Derseh L. Prevalence and associated factors of circumcision among daughters of reproductive aged women in the Hababo Guduru District, Western Ethiopia: A cross-sectional study. BMC Women’s Health. 2016;16(1):42. 10.1186/s12905-016-0322-6 27449648 PMC4957895

[28] Karmaker B, Kandala NB, Chung D, Clarke A. Factors associated with female genital mutilation in Burkina Faso and its policy implications. Int J Equity Health. 2011;10:20. 10.1186/1475-9276-10-20 21592338 PMC3112389

[29] Sakeah E, Debpuur C, Oduro AR, et al. Prevalence and factors associated with female genital mutilation among women of reproductive age in the Bawku municipality and Pusiga District of northern Ghana. BMC Women’s Health. 2018;18(1):150. 10.1186/s12905-018-0643-8 30227845 PMC6145319

[30] El-Dirani Z, Farouki L, Akl C, Ali U, Akik C, McCall SJ. Factors associated with female genital mutilation: A systematic review and synthesis of national, regional and community-based studies. BMJ Sex Reprod Health. 2022;48(3):169–178. 10.1136/bmjsrh-2021-201399 PMC9279756 35264420

[31] Fagbamigbe AF, Morhason-Bello IO, Kareem YO, Idemudia ES. Hierarchical modelling of factors associated with the practice and perpetuation of female genital mutilation in the next generation of women in Africa. PLoS One. 2021;16(4):e0250411. 10.1371/journal.pone.0250411 33891651 PMC8064566

[32] Mackie G, LeJeune J. Social dynamics of abandonment of harmful practices: A new look at the theory. Innocenti Working Paper No. 2009-06. Florence: UNICEF Innocenti Research Centre; 2009.

[33] National Institute of Statistics. ICF. Guinea Demographic and Health Survey (DHS V) 2016-18. Conakry: INS/Guinea and ICF; 2019.

[34] National Institute of Statistics and Demography (INSD). Burkina Faso 2010. Multiple indicator demographic and health survey (EDSBF-MICS IV). Ouagadougou (Burkina Faso): INSD and ICF International; 2012.

[35] National Institute of Statistics, Economic and Demographic Studies (INSEED). Demographic and health survey and multiple indicators in Chad 2014–2015. Calverton (Maryland): ICF International; 2016.

[36] Nnanatu CC, Atilola G, Komba P, et al. Evaluating changes in the prevalence of female genital mutilation/cutting among 0-14 years old girls in Nigeria using data from multiple surveys: A novel Bayesian hierarchical spatio-temporal model. PLoS One. 2021;16(2):e0246661. 10.1371/journal.pone.0246661 33577614 PMC7880428

